# Understanding American Indian/Alaska Native Students’ Barriers and Facilitators in the Pursuit of Health Professions Careers in Nebraska

**DOI:** 10.3390/genealogy8040133

**Published:** 2024-11-01

**Authors:** Keyonna M. King, Regina Idoate, Cole C. Allick, Ron Shope, Magdalena Haakenstad, Melissa A. Leon, Aislinn Rookwood, Hannah Butler Robbins, Armando De Alba, Sonja F. Tutsch-Bryant, Mariah Abney, Vanessa Hamilton, Patrik L. Johansson

**Affiliations:** 1 University of Nebraska Medical Center, Omaha, NE 68198, USA; 2 Institute for Research and Education to Advance Community Health, Washington State University, Seattle, WA 98101, USA; 3 Nebraska Indian Community College, Macy, NE 68039, USA

**Keywords:** American Indian, health professions education, under-represented minority, URM, health careers

## Abstract

The U.S. health care system presents American Indian/Alaska Native populations with inequitable challenges that result in some of the worst health outcomes in the country. The literature indicates that increasing the proportion of American Indian/Alaska Native health professionals can improve these health disparities. This study aimed to explore the severe under-representation of American Indians and Alaska Natives in Nebraska’s health professions workforce by examining barriers and facilitators in this population’s pursuit of health professions careers. We conducted demographic questionnaires and three talking circles with students in reservation and urban settings to better understand their lived experiences of pursuing health professions careers. We analyzed these qualitative data through content analysis and identified eight emergent themes—four barriers and four facilitators. These findings can inform the development of strategies to improve Indigenous education, research, and pathways that promote increased American Indian/Alaska Native representation in health care.

## 1. Introduction

American Indians and Alaska Natives (AI/ANs) are under-represented in the United States (U.S.) health professions workforce (Merchant and Omary 2010, p. 19). Comprising 2.9% of the U.S. population as of 2020, it is estimated that approximately 9.7 million people in the U.S. identified themselves as AI/AN alone or in combination with one or more other races (United States Census Bureau 2020). Yet, in 2021, only 0.3% of the 841,322 active physicians and 3.5% of the 194,510 total full-time faculty members at U.S. medical schools identified themselves as AI/ANs (Association of American Medical Colleges 2021). Only 6.8% of AI/AN students pursued a major in health sciences (United States Department of Education, National Postsecondary Student Aid Study 2016). While the percentage of AI/AN full-time faculty members has grown since 2016, the percentage of AI/AN active physicians has decreased although the AI/AN population continues to grow (Association of American Indian Physicians 2019). AI/ANs are also under-represented in other health professions, representing only 0.5% of registered nurses (Smiley et al. 2021; Nurse Practitioners Schools 2021), 0.3% of physical therapists (DataUSA n.d.; Association of American Medical Colleges 2021), 0.3% of physician assistants (National Commission on Certification of Physician Assistants 2022), and 0.5% of medical assistants (DataUSA n.d.).

The under-representation of AI/AN health professionals may contribute to the health disparities experienced by AI/ANs (Institute of Medicine 2003, pp. 38–48; Hulen et al. 2019; Skewes et al. 2020; Don 2022). Health disparities are defined as “a particular type of health difference that is closely linked with social, economic, and/or environmental disadvantage. Health disparities adversely affect groups of people who have systematically experienced greater obstacles to health based on their racial or ethnic group; religion; socioeconomic status; gender; age; mental health; cognitive, sensory, or physical disability; sexual orientation or gender identity; geographic location; or other characteristics historically linked to discrimination or exclusion” (Healthy People 2020). The COVID-19 pandemic has highlighted these disparities where AI/ANs were disproportionately affected by the pandemic compared to Whites (Hatcher et al. 2020, p. 1166). Our study examines barriers and facilitators for promoting health professions careers among AI/ANs living in Nebraska, a population that not only experiences multiple health disparities compared to Whites but also is severely under-represented in the state’s health professions workforce. Also, we provide recommendations to inform efforts to promote health professions careers among AI/ANs for which there are no existing publicly available data or published recommendations for best practices to increase AI/AN representation in the health professions workforce.

Five members of the research team (authors of this manuscript) identify as Native American who have participated in Western academic institutions and are a part of the community studied. This work is thus grounded in community episteme and draws from Indigenous values, knowledges, and ways of being that access other ways of knowing, such as Western theoretical and philosophical standpoints. All members of the research team identify with a group that is under-represented in the health professions. We were intentional about focusing on AI/ANs outside of the larger context of racial under-representation because we have published two other papers which focus on the under-representation of two other racial and ethnic groups—Black Americans (King et al. 2023) and Latinos (Johansson et al. 2020)—as part of the larger under-represented minorities in healthcare professions project that this manuscript stems from.

### 1.1. AI/AN Representation in Health Profession Careers

The National Institutes of Health and other agencies call for an increase in the representation of under-represented minorities in the health professions, including AI/ANs, as a means to address these health disparities. These calls are based on evidence that indicates AI/ANs and other under-represented minorities are more likely to practice in their communities and that racial concordance may increase patient adherence to treatment (Basco et al. 2010, p. 460; Cooper-Patrick et al. 1999, p. 583; Institute of Medicine 2003, p. 125; LaVeist and Pierre 2014, pp. 9–14; National Institutes of Health 2019). In addition, the Association of American Medical Colleges recognizes that AI/ANs are more likely to practice in rural areas (Association of American Medical Colleges 2014; Association of American Medical Colleges and the Association of American Indian Physicians 2018) where AI/ANs reside. As part of a solution to tackle health disparities in the AI/AN community, a critical need exists to increase the representation of AI/ANs in the health professions.

In Nebraska, AI/ANs are under-represented among health care professionals and experience alarming health disparities (Nebraska Department of Health and Human Services 2020). In 2022, 1.6% of the Nebraska population identified as AI/AN alone (United States Census Bureau 2022), but in 2019, AI/ANs represented only 0.1% of physicians (*N* = 4), 0.4% of physician assistants (*N* = 3), 0.2% of nurse practitioners (*N* = 2), 0.2% of dentists (*N* = 2), and 0.1% of pharmacists (Wehbi et al. 2020); moreover, there were no AI/AN physical therapists, occupational therapists, certified nurse midwives, or certified registered nurse anesthetists (Wehbi et al. 2020). This under-representation occurs in the context of glaring health disparities where the 73.1-year life expectancy for AI/ANs is 5.8 years shorter than that of Whites (Dwyer-Lindgren et al. 2022). Interestingly, there was no improvement in life expectancy for the AI/AN population from 2000 to 2019 although there was improvement across all other racial/ethnic groups. It is not clearly understood why these disparities exist in Nebraska regarding the number of AI/ANs in health professions careers. We could not identify any studies specific to Nebraska that examine barriers and/or facilitators to promote health professions careers in AI/ANs.

### 1.2. Barriers for Pursuing Health Professions Careers among AI/ANs

AI/AN health professions shortages have numerous root causes. Systemic racism manifested as the historical oppression and discrimination of AI/ANs that barred access to and/or full participation in higher education for generations has contributed to AI/ANs’ designation as historically under-represented groups (Brown University 2016). Additional barriers that contribute to disparity in the number of AI/ANs in the health professions include financial costs associated with higher education; lack of exposure to AI/AN health professionals and faculty who can provide culturally informed mentorship and support throughout students’ educational and early professional careers; limited academic preparation and support; challenges for first-generation students to maintain their ethnic identity and struggles to graduate from college; feelings of cultural isolation and loneliness (Association of American Indian Physicians 2019; Evans 2008, p. 205; Hollow et al. 2006, p. S65; Lopez 2010, p. 381; Sequist 2007, p. 20); and self-doubt (Sánchez et al. 2016, pp. 871–80).

### 1.3. Facilitators to Improve AI/AN Representation in Health Professions

Several facilitators to support AI/AN students in the pursuit of health professions have been noted in the literature; these include identifying students with motivation to serve Native peoples (Association of American Medical Colleges and the Association of American Indian Physicians 2018); introducing students at earlier ages to health professions careers; improving academic preparation through academic enrichment programs beginning in middle school and continuing through high school and college; expanding financing opportunities for health professions education such as additional scholarships, loan repayment programs, and tribal or private scholarships; providing mentoring by AI/AN health professionals and ongoing career development for AI/AN students (Association of American Indian Physicians 2019; Association of American Medical Colleges and the Association of American Indian Physicians 2018; Jackson et al. 2019, p. 70; Sánchez et al. 2016, pp. 871–80; Sequist 2007, pp. 820–30); creating support structures within academic institutions that address institutional racism and support racial equity to allow the integration of current and future AI/AN health professionals in safe spaces where they feel a sense of belonging (Sánchez et al. 2016, pp. 871–80); and having academic institutions encourage parental and community involvement in the education of AI/AN health professions students (Lopez 2010, pp. 381–91).

Our study aimed to identify ways to increase representation of AI/AN health professionals in a geographic region where there exists a gap in the literature on barriers and facilitators for promoting health professions careers among rural and urban AI/Ans. For the purpose of this paper, we defined rural AI/ANs as people or tribes who live in counties outside the boundaries of metropolitan areas (United States Department of Agriculture 2021).

## 2. Results

### 2.1. Participant Demographics

All demographic characteristics can be seen in Table 1. All study participants self-identified as AI/AN. The four participants in the high school talking circle were all 17 years old; one person identified as male, and three identified as female. The 15 participants in the two undergraduate student talking circles were aged 19 years to 34 years; 2 identified as male and 13 as female. Undergraduate student participants had a mean age of 24.9 years. Two out of the four high school students were affiliated with one of the four Nebraska Tribal Nations: the Omaha Tribe of Nebraska, the Ponca Tribe of Nebraska, the Santee Sioux Tribe of Nebraska, and the Winnebago Tribe of Nebraska, while 13 out of the 15 undergraduate students were affiliated with a Nebraska Tribal Nation. Among the high school students, two of the four participants were the first in their family to pursue a postsecondary college education. Among the undergraduate students, 8 of the 15 were the first in their family to pursue a postsecondary college education. None of the parents of either high school or undergraduate students had attended graduate school. Three of the high school students lived off and one student lived on a reservation, while 5 undergraduate students lived on a reservation and 10 lived off reservation.

### 2.2. Talking Circle Themes

The three talking circles yielded four themes related to barriers and four related to facilitators in AI/AN pursuits of health professions careers (Table 2). Perceived barriers in pursuing health professions careers included the following: (a) lack of preparation and support, (b) cost of college, (c) self-doubt, and (d) balancing priorities. Perceived facilitators in pursuing health professions careers included the following: (a) culturally relevant mentorship/student support, (b) exposure to learning opportunities, (c) self-belief, and (d) motivational mindset. During the qualitative data analysis, both coders in consultation with two senior investigators reached consensus in identifying the themes.

### 2.3. Barrier: Lack of Preparation and Support

A common barrier across all three groups in pursuit of health professions careers was a lack of academic and other support. One participant explained, “Nobody told me what classes I needed to take to get into (medical school)”. Without such guidance, many students reported that they did not take enough preparatory coursework in math, science, or writing to support them in meeting the academic requirements in health professions programs. Some participants reported that they did not take any science classes. As a result, the most talked about area of struggle was math. Some said that they “hated it” or “it stressed me out”. They described it as “real hard” or “like reading Chinese”. One participant said, “Math and science are the hardest subjects in school”. Participants wondered how math applies to a health profession. In general, participants expressed negative early experiences with math and science and a sense of feeling inadequately prepared for health professions studies as a result.

Participants also acknowledged the need to “have someone like a guidance counselor or someone to help”. One participant explained, “I always knew … I want to go to school. I want to be something better, but … I didn’t know how to do that”, referencing a lack of personal or professional support. This barrier was recognized by participants as “not having someone to show me or tell me what I was supposed to do”. Participants emphasized this lack of support noting, “You’re just really on your own” or “I have to do everything on my own, basically”. One participant said that there is “no one pushing you to do it, no one behind you trying to give you encouragement”. In addition to support from school, participants also expressed that there was a lack of support from their family. As one participant explained, “I tried to go and stay in school. But, my mom said, ‘No… . What you going to school for?’” Another poignantly stated, “some of our parents … just want you to be that good mom … you know, they want their daughter to be like good happy family, and sometimes that does mean… putting school on hold or … wait until those kids are out to school and then you can go back and go get that career, and so you … have to put your dreams on hold”.

### 2.4. Barrier: Cost of College

In addition to the lack of preparation and support, participants identified the cost of transportation, college supplies, living expenses, and tuition as a barrier to advancing into a health professions career. The prevailing perception among participants was that college is too expensive or that “school always costs a lot if it’s out of your own pocket, and a lot of us don’t have that money”. Participants emphasized the costs of loans, living in the dorms, having food or a meal plan, needing gas for their cars, textbooks for class, and a computer to complete assignments, noting “it’s just too much” and “it’s hard enough to pay for classes”. As one participant said, “I don’t already make enough to go to school, but I have to make it work to get this far”. Participants also expressed that it was a challenge to find resources to cover the costs of college. One participant put it this way: “I think going farther (in school) is going to take away from my kids financially”. In addition, the costs of daily living, including the cost of utilities, groceries, insurance, car payments, gas, diapers, wipes, and baby formula, made it challenging to take on the financial cost of school. One participant said, “I have five kids… .I have to buy food, Pampers, whatever they need, their softball games, their extracurricular things all cost money, so it’s kind of hard for me to stay in school”.

### 2.5. Barrier: Self-Doubt

Self-doubt surfaced in multiple participants’ descriptions of their lived experiences navigating the pursuit of health professions. Many doubted that their grades would meet admissions standards, some doubted the quality of their work, and others did not believe they could reach the goal of becoming a health care professional. Others doubted they could succeed in math or science courses while others questioned whether or not they had the required self-discipline to successfully complete a health professions career track. One participant said, “Everybody always says you have to have good grades to go to college … and I don’t think my grades are up there for college. I doubt myself a lot about it”. Some participants expressed their doubts in more generalized statements such as “Math and science are the hardest subjects in school and most people don’t think that they are good at it”, while others shared more personal statements such as “I’m not smart enough to do this” or “I always have to have a little more confidence in myself”.

Observing that self-doubt influenced their interests, inquiries, and initiative in pursuing health professions, participants reported a need for “confidence building” or to learn how to “speak and have confidence in themselves”. Many expressed “feeling judged” or “intimidated” by schools, counselors, peers, and/or teachers. One participant explained, “If I didn’t have the confidence … I’m not gonna deal with it. … it just felt easier not to deal with it, just to move to something … easier”. There was also a struggle with speaking up in class when something was not understood. One participant put it as follows: “It’s not always easy to go up to a professor and say I don’t really understand this”. Although many recognized the struggle as normal, they noted, “a lot of Natives … are shy and they don’t know how to speak up”, “… or how to say that they need help”.

### 2.6. Barrier: Balancing Priorities

Participants referenced various challenges in balancing the pursuit of health professions careers with other life responsibilities such as caring for relatives, parenting, traditional activities (e.g., tribal responsibilities and ceremony), health concerns, personal reasons, and other interests. This theme resonated among many participants especially when discussing the topic of school. Participants acknowledged the balance between employment and academics. One participant stated, “You gotta balance between work and school at the same time” or “Finish your work then worry about what’s going on at home”. Another participant indicated that sometimes work is prioritized over school, “… you’re not encouraged to go to school” and quoted family members saying, “You have to go to work you better get out there and work”. Participants also noted the importance of traditional activities, stating, “we’re always praying, we’re always doing ceremonies and we’re always there for each other” and argued, “We need to be more… traditional, honestly”. Yet, they agreed that the pursuit of a health professions career requires some prioritization of school. This was expressed as a challenge in multiple participant comments such as, “You have all these different things going on after school, just pulling at you”. Noting the struggle to balance academic and work life, one participant said, “There’s time between work to do homework, but nobody wants to do that, ‘cause you’re trying to focus on getting your money, saving up your money”. Many participants also noted distracting interests or “other things to do” that diverted their attention from school, such as “a lot of bad habits … drug use and drinking, staying up late, poor eating habits, and exercise and stuff like that” that divert their attention from school. Participants also noted that meeting family responsibilities is an important priority in life that could impede their time commitment and dedication to school. The following lived experiences were given as examples: “I tried to stay in school, but then I was a young mom…and then I just wasn’t allowed in school because I was pregnant;” “I also have siblings that look up to me as a kind of like a mother figure…I go to them more than I go to school. … I’ll help them out before I even think about going to school”.

### 2.7. Facilitator: Culturally Relevant Mentorship/Student Support

Students in the three focus groups indicated the importance of culturally relevant mentorship and student support in promoting health professions. One participant said, “Native students are losing their culture”. Overall, participants noted little integration of traditional teachings and practices from across disciplines and cultures and identified this as a barrier. Participants who reported receiving support acknowledged their Creator, relatives, peers, mentors, and teachers as facilitators in their pursuit of health professions. One participant stated, “When we pray, we pray for everybody that has our blood, and the power of prayer is really strong, and if you believe and how much you believe in the Creator, it’s gonna take you places”.

### 2.8. Family/Peer Support

In addition to spiritual support, participants noted familial support as a facilitator. One participant asserted, “I know my parents always told me I can do this … and they had a lot of confidence in me”. Participants who received support from their family members explained this as a strong source of motivation. One example of how participants described this include, “my mom. She’s in medical school. She makes me want to just go on with my own thing”.

Peers were also noted as another form of support. Participants reported that the “like-minded” support they received from their peers helped them with their studies. One participant explained that when they work together with peers they “can relate and … it’s more comfortable”. Teachers and mentors were also recognized as facilitators. One participant shared, “I have to have somebody telling me … I’m okay … You’re all right. You’re doing it the right way. I have to constantly have that, otherwise, I’ll just stop”. Encouragement from teachers and mentors was acknowledged as a strong facilitator. Additionally, we noted teachers and mentors who offer “more interaction, less than just writing something down, take more time to explain … and take the time to help” demonstrated cultural responsiveness.

### 2.9. Facilitator: Exposure to Learning Opportunities

Another consistently mentioned form of support was exposure to an academic- and career-focused pathway and other enrichment programs that expose students to subject matters, academic institutions, and educational/work opportunities to learn and grow toward a health professions career. Participants emphasized the importance of “different schools”, “experiences”, and “exposing (future health care professionals) to more diversity … more opportunities … other places”. Participants spoke highly of their experiences in workforce development programs. One individual explained that academic enrichment opportunities are helping students with “getting used to the outside world” and “start exposing them to more diversity so they’re ready when they go out”. One participant explained that these programs can help “Native kids” realize that “there’s more opportunities when you go and network in other places” because “not everything is always just gonna be a rez [reservation]”. Participants emphasized and suggested that without involvement in a pipeline program many students would not pursue a health professions career, saying, “If you don’t know about it, you won’t do anything” and hailing the support that comes from learning that “there’s this program for this. You can do that”.

### 2.10. Facilitator: Self-Belief

Students noted the importance of believing in themselves and believing in their ability to succeed in meeting goals, performing tasks, and overcoming academic challenges in their pursuit of health professions careers. One participant noted that “believing in yourself” is “a really big thing”, and another affirmed the need to “believe in yourself that you can do it”. Participants emphasized the importance of believing in their ability to succeed in their pursuit of health professions careers. One participant summarized this attitude: “If you don’t believe in yourself, you’re not gonna really feel like you could do it”. Another participant elaborated, “If you believe in yourself that much, it’s gonna take you places”. Many participants demonstrated their belief in their ability to study, to work, and to do what it takes to achieve a health professions career. Below are examples of self-belief statements among participants: “I really like science. I think my grades are good enough;” “I feel like I get along with others in…the nursing field. …I could sit and just listen and converse because I love talking about (it) … I’m a science nerd;” “I’m not scared to ask questions …if I want to know something. I’ll just ask”.

### 2.11. Facilitator: Motivational Mindset

When asked what advice they would give other AI/ANs pursuing health professions careers, many participants emphasized the importance of a “motivational mindset”. Below are some examples of their advice: “Give yourself time … and make those day-by-day goals. And once you know how to do that day by day, then start week. And then, pretty soon, it’ll turn into months”; “Just keep consistent with yourself … and the biggest thing: pray because you can do it”; “You gotta make it work if you, if that’s what you want, that’s what you gotta do”; and “You just gotta put yourself out there to get what you need to get and do what you need to do to get to where you wanna get to”.

Participants often referenced inspirational motivators for pursuing a health professions career such as family, finances, community, spirituality, and vision. For example, one participant was motivated by the desire to research cancer because it had affected their relatives, saying, “We have cancer, so I’m doing cancer research for my internship”. Participants referred to this inspiration as “just people having the mindset to do it”. Below are some additional examples of how participants illustrated a motivational mindset: “Just praying, wanting better for yourself and others around you. My family comes from a really cultural background … … you just wanna do it for them too, say they’re proud of you, and for yourself, for everything that we pray for. I just want to do better for myself, for everyone who prayed for me, and just for how far I’ve already made it”.

## 3. Discussion

Our study examined barriers and facilitators for promoting health professions careers among urban and rural AI/AN high school and tribal college students. We employed tenets of a community-based participatory research approach grounded in Indigenous research methodologies through an American Indian-led research team using talking circles to conduct this study. We identified four barriers (lack of preparation and support, cost of college, self-doubt, and balancing priorities) and four facilitators (culturally relevant mentorship/student support, exposure to learning opportunities, self-belief, and motivational mindset) to pursuing health professions careers. The novelty of our study lies in the gap in the literature on this topic among rural and urban AI/ANs in Nebraska, a population that experiences striking health disparities in comparison to non-Hispanic Whites (Nebraska Department of Health and Human Services 2020).

Our findings confirm an existing body of evidence surrounding factors influencing the representation of AI/ANs among health professionals. All four thematic barriers we identified have been described in the literature: a lack of academic preparation, cost of higher education, competing priorities and facilitators for pursuing health professions careers (Association of American Indian Physicians 2019; Evans 2008, pp. 205–17; Hollow et al. 2006, p. S65; Lopez 2010, p. 381; Sequist 2007, pp. 20–30), and self-doubt (Sánchez et al. 2016, p. 877). Identified facilitators in our study also described in the literature include the following: identifying students with motivation to serve Native people (Association of American Medical Colleges and the Association of American Indian Physicians 2018); introducing students at earlier ages to health professions careers; improving academic preparation through academic enrichment programs; expanding financing opportunities for health professions education; and engagement with AI/AN health professionals who can provide culturally informed mentorship and support (Association of American Indian Physicians 2019; Association of American Medical Colleges and the Association of American Indian Physicians 2018; Jackson et al. 2019, p. 70; Sánchez et al. 2016, pp. 871–80; Pavel 2013; Sequist 2007, pp. 29–30). The importance of culturally informed mentorship by AI/AN health professionals is also consistent with findings by Waterman et al. (2018) that underscore the importance of having Indigenized education programs developed by Indigenous communities that value Indigenous perspectives and knowledge. Our study also showed that participants identified self-belief, defined as importance of believing in themselves and believing in their ability to succeed in meeting goals, performing tasks, and overcoming academic challenges in their pursuit of health professions careers as playing a major role in promoting these careers in this population. The latter finding is consistent with research from Sánchez et al., which indicates that self-doubt is a barrier for AI/ANs to pursue health professions careers (Sánchez et al. 2016, pp. 871–80).

Our findings are also corroborated by Indigenous scholars who have published extensive work related to the under-representation of AI/AN students in postsecondary education. These scholars also discuss how colonial schooling practices have strategically influenced access to and participation in postsecondary educational opportunities. For instance, Lomawaima and McCarty (2006) developed a theoretical framework labeled “the safety zone” to explain perpetual conflict over the issue of cultural difference in educational settings. Educational policies and practices have traditionally reflected the federal government’s attempt to make a distinction between “safe” and “dangerous” Indigenous beliefs and practices. This led to the sanctioning of stories, arts, and crafts that are profitable and “safe”, and deemed “dangerous” practices and beliefs as AI languages and spiritual practices, including music and songs intimately connected to religious experiences. Ultimately, these policies and practices led to the extinction of many significant Indigenous beliefs and practices in educational settings. The assimilative attitudes of colonial schooling practices did not appear overnight. Rather, they percolated from centuries of U.S. sanctioned policies—including boarding schools and Indian adoption projects—aimed at the erasure of Native American cultures (Graham 1998, p. 1).

These practices also led to AI/ANs resisting federal policy and shaping their own approach to educating AI/AN children. In addition, Shotton and Minthorn also noted the limited research funding leveraged for studying Indigenous students and excluding their research interests from projects that typically address diversity in postsecondary education (Shotton and Minthorn 2018). AI/AN voices and perspectives are consistently left out of educational policy making, and schools lack acceptance in embracing diverse learning modes that could attract AI/AN students (Shotton and Minthorn 2018; Waterman et al. 2018; Lomawaima and McCarty 2006). Therefore, it is important to reconcile these differences in educational settings, particularly healthcare education, to ensure Indigenous and non-Indigenous faculty and students are socially accountable for developing and implementing education and healthcare mandates as it relates to Indigenous health beliefs (Burm et al. 2024).

Unlike findings in the literature (Association of American Indian Physicians 2019; Evans 2008, pp. 205–17; Hollow et al. 2006, p. S65; Lopez 2010, pp. 381–87; Sequist 2007, pp. 20–30), participants did not note social or cultural isolation as a barrier; however, they did note culturally relevant mentorship/student support as a facilitator. All of the high school student participants took part in the Youth Enjoy Science (YES) program, a culture-based academic enrichment program where all identified as AI/AN and were all enrolled in the Native Indigenous Centered Education Program of Omaha Public Schools. Furthermore, all of the undergraduate students were enrolled at Nebraska Indian Community College, an institution focused on preserving and revitalizing the cultures of the Omaha and Santee people, where the majority of students are AI/AN. Therefore, isolation from AI/AN culture and/or other AI/ANs may not have emerged as a theme, underscoring the important roles that culture-based academic enrichment programs and tribal colleges play in addressing this barrier. In addition, our finding that peer support from a cohort of fellow AI/AN students going through the same academic experience represents a facilitator promoting health professions careers in this population is also consistent with the literature (Lopez 2010, pp. 381–87) and speaks to the importance of AI/AN specific culture-based academic enrichment opportunities.

It is important to contextualize our results to the socioeconomic disparities experienced by AI/ANs nationally and in Nebraska. At 25.9%, AI/ANs experience the highest poverty rate among all races and ethnicities in the U.S. (Kaiser Family Foundation 2021). In Nebraska, with a poverty rate of 21.1%, AI/ANs are two-and-half-times more likely than Whites (8.1%) to live below the poverty level (Kaiser Family Foundation 2021). The combination of poverty and disparities in education and health have created an urgent need for AI/AN health professionals while concurrently contributing to their under-representation in these fields. Although AI/AN students who are citizens of federally recognized tribes are born with inherent rights to health care and education via formal treaties signed with the federal government, these programs are severely underfunded (Warne 2007, pp. 11–19). Subsequently, interventions seeking to improve the representation of AI/ANs in health professions need to create strategies that address the significant social, demographic, cultural, academic, and financial obstacles faced by many AI/AN students (Acosta and Olsen 2006, pp. 863–70). Such programs should address social determinants of health, including supporting whole-person health, developing culturally informed health and well-being programs, and investing in expanding access to affordable housing, transportation, and career counseling (Association of American Indian Physicians 2019).

Tribal colleges and universities, such as Nebraska Indian Community College, are uniquely positioned to tackle challenges faced by AI/AN students, as at their core they were created to meet both the cultural and the higher educational needs of AI/AN students. They are significantly regenerating tribal culture and education, two priorities for AI/AN communities, while grounding their educational philosophies and approaches in cultural traditions (American Indian Higher Education Consortium n.d.). Acknowledging a mix of culture, language, heritage, clan structure, and governance that is unique to the identity of each AI/AN sovereign nation (De Long et al. 2016, p. 104), tribal colleges and universities have been instrumental in influencing student success and persistence in their pursuit of higher education at both tribal and nontribal institutions. For example, the Salish Kootenai College’s nursing program has led the nation in graduating AI/AN registered nurses since its establishment in 1988 by providing individualized education and a supportive learning environment to AI/AN nursing students that address health, well-being, and cultural identity (Salish Kootenai College 2020).

Tribal colleges and universities can be especially supportive of AI/AN students in their pursuit of health professions careers because they address socioeconomic challenges that students face by providing services that go beyond academic support (Bryan 2019, pp. 49–62). Some of the services they offered include personal/career counseling, mentoring, tutoring, wellness programs, child care, laptop lending, and transportation/housing assistance; providing student mentorship from faculty and staff (many of whom are AI/AN) when transitioning to a mainstream four-year institution; integrating culture and practice into coursework including history, language, and values; and assisting students in adjusting to the academic environment of postsecondary education before having to adjust to the social environment of a four-year mainstream institution (Bryan 2019, pp. 49–62).

### Limitations

This study had limitations. The sample of participants within each age cohort was small. We used a convenience sample of Nebraska rural and urban AI/AN high school and undergraduate students with an interest in pursuing a health professions career. The individuals who participated in our study may not be representative of all rural and urban AI/AN student, a general limitation of qualitative inquiry. In addition, most participants were students at Nebraska Indian Community College, a tribal college, and the majority of participants reported tribal affiliation with a Nebraska Tribal Nation, which may have influenced results. The four tribes of Nebraska identified in our study as the Omaha Tribe of Nebraska, the Ponca Tribe of Nebraska, the Santee Sioux Tribe of Nebraska, and the Winnebago Tribe of Nebraska is not representative of all tribal nations that resided in modern-day Nebraska. Historically, the tribal nations represented also includes the Arapahoe, Cheyenne, Delaware, Ioway, Kaw, Kickapoo, Meskwaki, Otoe-Missouria, Pawnee, Sioux, and Wichita (Swanton 2003). A major limitation of talking circle groups is that some participants may be more able to express their ideas than others in a group setting. Although results from our study provide descriptive narrative, generalization to the views of other urban or rural AI/ANs in Nebraska on pursuing health professions careers is not assured.

## 4. Materials and Methods

### 4.1. Theoretical Framework

This project is relationally organized. Indigenous studies usually adopt a phenomenon-driven approach to understand the issues, actors, and their behaviors in a specific setting. This approach to theory makes indigenous theory realistic and descriptive. Indigenous theory (Sinclair et al. 2009; Nabigon 2006; Poonwassie and Charter 2005; Solomon and Wane 2005, p. 52; Graveline 2004) results in theory operating close to action (where decisions are made), making it especially attentive to the complex motives of different actors and the related power dynamics and underlying forces (Bruton et al. 2021). Therefore, we identified the community-based participatory research approach as appropriate for the work presented in this paper.

The National Congress of American Indians has identified the community-based participatory research approach as an effective approach to conduct research with Tribal communities (National Congress of American Indians 2009). The community-based participatory research approach is defined as a collaboration between academia and community in examining key community issues and developing appropriate interventions to address the issues (Israel et al. 2013). We used principles of the community-based participatory research approach as our theoretical framework in this study by engaging members of the participating tribal college in all phases of the project to center our work on community priorities (Israel et al. 1998, pp. 173–202). In light of the literature showing that the presence of AI/AN project leads enhances AI/AN participation in research (Buchwald et al. 2006, pp. 648–51), our diverse research team included AI/AN lead investigators and AI/AN research assistants/students pursuing health profession careers.

### 4.2. Design

In this qualitative study, we used purposive sampling (purposefully selecting individuals, groups, and settings based on identity, geographical location, and experience with health professions career paths) to recruit AI/AN high school students exposed to health sciences and undergraduate student participants who expressed interest in pursuing a health professions career (Creswell and Poth 2018). All participants responded to a brief demographic questionnaire and engaged in a talking circle that gathered more in-depth information. Talking circles, a long-standing tradition still employed by numerous AI/AN tribes, is a group process that invites those in the circle to participate in dialogue involving decision-making and problem-solving (Struthers et al. 2003, p. 1094). We chose talking circles as they are an effective way to remove barriers of communication, allowing people to express themselves freely, and they have proven successful in health education and promotion activities with AI/ANs (Struthers et al. 2003, pp. 1094–15). We digitally recorded and transcribed the talking circles. This study received institutional review board approval (IRB #0192-17-EP) from the University of Nebraska Medical Center and the Nebraska Indian Community College. We sought parental consent for minors in writing.

### 4.3. Measures

Demographic Questionnaire. Our demographic questionnaire collected data on gender, race, ethnicity, tribal affiliation, residence by geography (i.e., on reservation or off reservation), education, and parental education.

Interview Guide. We used a semi-structured interview guide to assess barriers and facilitators to pursue health professions careers in the talking circles (Johansson et al. 2020, pp. 1–15). We presented participants with a list of barriers identified in the scholarly literature as challenges to under-represented minority students in pursuing health professions careers along with the option to name other challenges not listed (Johansson et al. 2020, pp. 1–15).

### 4.4. Participants and Recruitment

Study participant inclusion criteria were self-identification as AI/AN and being at least 14 years or older with an expressed interest in pursuing a health professions career. Participants were enrolled in the Youth Enjoy Science program, a research education program at the University of Nebraska Medical Center for AI/AN high school students (University of Nebraska Medical Center 2020) or at Nebraska Indian Community College, a tribal college serving the people of the Omaha and Santee Dakota Nations (Nebraska Indian Community College 2020). We conducted three talking circles, one with high school students and two with undergraduate students. High school students were recruited through a flyer, in-person announcements to the students made by the Youth Enjoy Science program manager, and recruitment letters sent to the parents of the students. An administrator at Nebraska Indian Community College recruited the undergraduate students through in-person announcements, an email message, and a recruitment flyer.

### 4.5. Procedure

Informed signed parental consent for minors and informed signed consent from participants 19 years and older was obtained prior to the start of the talking circles and distribution of the brief demographic survey. Participants were asked to complete the demographic survey prior to the start of the talking circles. The same AI/AN facilitator moderated each talking circle and engaged participants in discussions and storytelling related to their lived experiences, beliefs, values, and expectations specific to the barriers and facilitators involved in seeking a health professions career (Tolley et al. 2004). The moderator opened each talking circle with a prayer. In addition to the moderator, one or two research team members attended the talking circles for note-taking and logistical support. The talking circles were digitally recorded, ranging from 60 to 90 min, and mean duration of the talking circles was 75 min. Participants received a USD 20 gift card for taking part in a talking circle. The talking circle with high school students was held in a private conference room on the campus where the YES program took place. The other two talking circles with undergraduate students were held in private conference rooms on the campuses of Nebraska Indian Community College. We used a card-sorting activity to facilitate the talking circle discussion, a methodology successfully employed to examine barriers and facilitators in promoting health professions careers among Latinx students (Johansson et al. 2020, pp. 1–15; Tolley et al. 2004). We printed each of the following 13 barriers for pursuing health professions careers on a single-sided, standard letter-sized paper and then taped the list in large font to the wall:

1. I have a difficult time connecting with someone who can help me enter a health profession (if you are in high school, this may be someone like a guidance counselor);
2. I don’t feel academically prepared to be in a health profession;
3. I don’t feel I am strong enough in math and science to be in a health profession;
4. My grades are not good enough to be in a health profession;
5. I do not feel like I belong with others who are seeking a health professions career;
6. I face discrimination in my school which makes it difficult to pursue health professions education;
7. I feel there are better jobs/careers out there;
8. I don’t think I could afford a health professions education;
9. My parents are unaware of the requirements for pursuing a health professions career;
10. My parents are not supportive of me pursuing health professions education;
11. I feel that I have to start working to financially support my family instead of spending more years in school;
12. There is a lack of exposure to opportunities in health professions careers;
13. I have other reasons that are not listed.

We provided each participant with five sticky dots and asked them to place one dot onto 5 of the 13 response options that we had posted on a wall in the room where we conducted the talking circle. Following review of the barriers and challenges, participants were asked open-ended questions regarding the leading barriers they listed and what they had done or could do or what helped them or could help them to overcome these barriers.

### 4.6. Data Analysis

For the demographic survey, we used descriptive statistics, specific counts, and percentages for categorical responses and mean values for age. An AI/AN investigator facilitated the identification of themes and codes through a group process with other members of the research team which included members reading and reviewing the transcripts from the talking circles. Three members of the research team independently identified emergent themes. Disagreements in coding were discussed until a consensus was reached for major themes that became a codebook, identifying codes with text examples from participants’ responses that established properties of each code. The codebook was entered into NVivo 12 (QSR International Pty Ltd. 2018). To ensure reliability, two coders independently read each transcript of the three talking circles, which they coded line-by-line. In identifying emergent themes, the coders discussed any disagreements with senior members of the research team until they reached consensus, and the themes were developed into a codebook. To validate our findings, two AI/AN undergraduate students from one of the recruitment pools reviewed the transcripts and the themes offering more context and clarifications of the data that helped confirm the accuracy of our results (Creswell and Poth 2018).

## 5. Conclusions

We studied barriers and facilitators for promoting health professions careers in an understudied population: rural and urban AI/AN students in Nebraska enrolled in high school or a tribal college. The novelty in our work stems from the presentation of voices of rural and urban Nebraska AI/AN students interested in health professions, a constituency in addressing health disparities in their communities, whose perspectives have not been previously described in the literature. We believe the identified barriers can be addressed in academic institutions nationally by partnering with AI/AN communities and leveraging the facilitators as a means of addressing some or all of the barriers. Our recommendations, based on our research, to strengthen AI/AN representation in health professions, align with those of the existing literature and include the following:

- Develop and deliver academic enrichment activities to strengthen competencies in science and math beginning in elementary school and continuing through high school;
- Provide AI/AN students with substantial financial support in terms of tuition reimbursement, scholarships, or work study jobs;
- Provide AI/AN students with mentoring and internship opportunities from AI/AN health professionals at the high school and undergraduate levels;
- Recruit and retain AI/AN faculty and staff to work in institutions that promote health professions careers;
- Ensure that there are AI/AN socially and culturally relevant places and supports at the high school, undergraduate, and health professions school levels. This could include but is not limited to establishing a multicultural affairs office, an Indigenous medicine garden, and offering curriculum, courses, or extracurricular activities taught by culturally congruent mentors/instructors linked to AI/AN culture and traditions;
- Develop and deliver interventions promoting health professions careers in this population that include components that promote self-belief and integrate AI/AN culture(s) in the curricula, an approach used by tribal colleges and universities;
- Partner with AI/AN serving programs and institutions (e.g., tribal colleges and universities) by adapting tenets already employed to address social determinants of health to serve AI/AN students.

This study can support future efforts to develop and deliver more comprehensive interventions that promote participation in health professions among rural and urban AI/AN students. Such interventions will be most successful if they are developed and offered in collaboration with intergenerational and inter-tribal students, educators, and health care professionals. Recognition of the AI/AN student perspective presented in our study and implementation of our recommendations requires an institutional, tribal, and personal commitment to strengthening AI/AN representation in health professions. It is our hope that this study will strengthen commitments to these efforts and inform the development of novel strategies to improve Indigenous education, research, and pathways to a more diverse health care workforce. Increasing representation of AI/AN health professionals is a critical step in addressing health disparities in AI/AN communities in Nebraska and across the United States.

## Figures and Tables

**Table 1.** Demographic characteristics of talking circle participants.

Demographic	Category	High School		Undergraduate		Total	
		*N*	%	*N*	%	*N*	%
Age	Less than 19 ^a^	4	100	0	0	4	21
	19–25	0	0	9	60	9	47
	26–30	0	0	4	27	4	21
	31–35	0	0	2	13	2	11
Gender	Female	3	75	11	73	14	74
	Male	1	25	4	27	5	26
Tribal affiliation	Nebraska Tribal Nations ^b^	2	50	12	80	14	74
	Other Tribal Nations ^c^	2	50	3	20	5	26
Parent/guardian highest level of education	High school	2	50	8	53	10	53
	Some college	2	50	3	20	5	26
	College graduate	0	0	4	27	4	21
	Graduate school	0	0	0	0	0	0
Residence	Live on reservation	3	75	5	33	8	42
	Live off reservation	1	25	10	67	11	58

Note. Mean age for high school participants = 17.0. Mean age for undergraduate participants = 24.9.

a The age of majority in the state of Nebraska is 19 years old.

b Represents participants who identified as at least one of the following four Nebraska Tribal Nations: the Omaha Tribe of Nebraska, the Ponca Tribe of Nebraska, the Santee Sioux Tribe of Nebraska, and the Winnebago Tribe of Nebraska.

c Other Tribal Nations represented in this study are the Cherokee, Navajo, Rosebud Lakota, Sioux, Sisseton Wahpeton Oyate, and Yankton.

**Table 2.** Talking circle themes and definitions.

Barriers	Definition
Lack of preparation and support	Insufficient preparedness for a health professions career track (e.g., lack of family/teacher support and lack of coursework offered/completed successfully)
Cost of college	Insufficient resources (e.g., transportation, books, and tuition) necessary to cover the costs of college programs leading to health professions careers.
Self-doubt	Doubts in themselves (e.g., doubts that their grades will meet standards, doubts in the quality of their work, doubts that they can succeed in math/science, doubts in self-confidence, and doubts in self-discernment) in their pursuit of health professions careers.
Balancing priorities	Challenges in balancing school with other competing priorities in life such as parents, kids, traditional activities, health concerns, personal reasons, and other interests.
Facilitators	Definition
Culturally relevant mentorship/student support	Supported by culturally relevant mentors, teachers, and peers who can help them enter a health profession.
Exposure to learning opportunities	Supported by academic- and career-focused programs that expose them to subject matter, academic institutions, and educational/work opportunities to learn and grow.
Self-belief	Importance of believing in themselves and believing in their ability to succeed in meeting goals, performing tasks, and overcoming academic challenges in their pursuit of health professions careers.
Motivational mindset	Motivators for pursuing a health professions career: family, community, money, spirituality, and vision.

## Data Availability

The datasets presented in this article are not readily available because the data include identifying information and may jeopardize the anonymity of participants. Requests to access the datasets should be directed to the corresponding author.
